# Functional differences of Toll‐like receptor 4 in osteogenesis, adipogenesis and chondrogenesis in human bone marrow‐derived mesenchymal stem cells

**DOI:** 10.1111/jcmm.16506

**Published:** 2021-05-03

**Authors:** Fatemeh Khodabandehloo, Reza Aflatoonian, Zahra Zandieh, Farzad Rajaei, Forugh‐Azam Sayahpour, Marjan Nassiri‐Asl, Mohamadreza Baghaban Eslaminejad

**Affiliations:** ^1^ Department of Molecular Medicine Qazvin University of Medical Sciences Qazvin Iran; ^2^ Department of Endocrinology and Female Infertility Reproductive Biomedicine Research Center Royan Institute for Reproductive Biomedicine ACECR Tehran Iran; ^3^ Department of Anatomy School of Medicine Iran University of Medical Sciences Tehran Iran; ^4^ Cellular and Molecular Research Center Research Institute for Prevention of Non‐Communicable Disease Qazvin University of Medical Sciences Qazvin Iran; ^5^ Department of Stem Cells and Developmental Biology Cell Science Research Center Royan Institute for Stem Cell Biology and Technology ACECR Tehran Iran; ^6^ Department of Pharmacology and Neurobiology Research Center Shahid Beheshti University of Medical Sciences Tehran Iran

**Keywords:** BM‐MSCs, differentiation, toll‐like receptor 4, Wnt5a

## Abstract

Multipotent human bone marrow‐derived mesenchymal stem cells (hMSCs) are promising candidates for bone and cartilage regeneration. Toll‐like receptor 4 (TLR4) is expressed by hMSCs and is a receptor for both exogenous and endogenous danger signals. TLRs have been shown to possess functional differences based on the species (human or mouse) they are isolated from therefore, the effects of knockdown of TLR4 were evaluated in humans during the differentiation of MSCs into bone, fat and chondrocyte cells in vitro. We investigated the expression profile of TLR4 during the differentiation of hMSCs into three different lineages on days 7, 14 and 21 and assessed the differentiation potential of the cells in the presence of lipopolysaccharide (LPS, as an exogenous agonist) and fibronectin fragment III‐1c (FnIII‐1c, as an endogenous agonist). TLR4 expression increased following the induction of hMSC differentiation into all three lineages. Alkaline phosphatase activity revealed that FnIII‐1c accelerated calcium deposition on day 7, whereas LPS increased calcium deposition on day 14. Chondrogenesis increased in the presence of LPS; however, FnIII‐1c acted as a reducer in the late stage. TLR4 silencing led to decreased osteogenesis and increased adipogenesis. Furthermore, Wnt5a expression was inversely related to chondrogenesis during the late stage of differentiation. We suggest that understanding the functionality of TLR4 (in the presence of pathogen or stress signal) during the differentiation of hMSCs into three lineages would be useful for MSC‐based treatments.

## 1 INTRODUCTION

Multipotent adult mesenchymal stem cells (MSCs) are adherent, fibroblast‐like and non‐hematopoietic cells, that have the capability to self‐renew and differentiate into three lineages including osteoblasts, chondrocytes and adipocytes.^1^, ^2^ It is well known that MSCs have a role in the regulation of proliferation, migration, differentiation and immunomodulation of cells.^3^, ^4^ It is necessary to understand how these functions are regulated by MSCs. The expression of TLRs was observed on different types of cells.^5^, ^6^ TLRs are also expressed on stem/progenitor cells and have been shown to control the survival, proliferation, differentiation and migration of these cells.^7^ Therefore, it is possible that MSCs perform many of their functions through TLRs.^8^, ^9^, ^10^, ^11^ TLRs are type‐I transmembrane glycoproteins comprising two domains, the leucine‐rich repeat (LRP) domain that can identify pathogen‐associated molecular patterns (PAMPs) and the Toll/IL‐1 receptor (TIR) domain that activates adaptor molecules including myeloid differentiation primary response 88 (MYD88), TIR domain receptor‐associated protein (TIRAP), TRIF‐related adaptor molecule (TRAM) and TIR domain‐containing adaptor protein inducing INF‐β (TRIF).^12^ Finally, nuclear factor kappa B (NF‐kB) and mitogen‐activated protein kinase (MAPK) are activated that enhance the production of proinflammatory cytokines.^13^

In addition to PAMPs (such as lipopolysaccharides),^13^ TLRs are also activated in the presence of heat‐shock proteins (HSPs), S100 proteins and extracellular matrix fragments that named damage‐associated molecular patterns (DAMPs) (such as fibronectin). DAMPs are released upon alteration in the structures of tissues, cellular stress or tissue damage.^14^, ^15^, ^16^ The FnIII‐1c is a Type III domain of fibronectin that, as a component of extracellular matrix molecules (ECM) can activate TLR4 signalling. Mechanical injury and tissue damage could drive the fate of hMSCs via TLRs.^17^ TLRs when triggered have a regulatory role in immune regulation, migration and the recruitment of MSCs in damaged tissues.^10^ We investigated how the presence of pathogens and stress/danger signals affects the signalling pathway of TLR4 during the differentiation of hMSCs into three different lineages. The role of Wnt5a in response to inflammation^18^ and differentiation^19^ in MSCs has been shown previously. The Wnt5a acts via the non‐canonical pathway and is involved in osteogenesis.^20^ The WNT5A/FZD4/JNK pathway is effective in inducing osteogenesis in mouse bone marrow (BM)‐derived MSCs via mechanical loading.^21^ Recently, it has been shown that the release of FnIII‐1c is controlled by mechanical forces and proteolysis.^17^ It was recently clarified that TLR4 performed its role in osteogenesis via Wnt5a in mouse BM‐MSCs,^22^ but its role in humans is not known.

Studies have not yet determined a relationship between the pattern of TLR4 expression (in the presence and absence of endogenous and exogenous ligands) and the pattern of gene expression during differentiation of MSCs into fat and cartilage cells. Moreover, the effect of FnIII‐1c as a stress signal on the differentiation potential of human MSC into bone, fat and chondrocyte cells is unknown.

In the present study, we tried to elucidate the molecular mechanisms and functions of TLR4 during the differentiation of human MSCs into three lineages. First, we studied expression patterns of TLR4 during differentiation into the three lineages and then created a model for inducing pathogen‐related and tissue damage‐related stress conditions through the activation of TLR4 via LPS and FnIII‐1c, respectively. Second, the expression of genes during differentiation of MSCs into all three lineages in the presence of both ligands was investigated. Given that so far there is no report on the effect of TLR4 knockdown on the differentiation capabilities of human MSCs into any of the three lineages, the function of TLR4 was evaluated by its knockdown in MSCs during differentiation into the respective lineages. In addition, we also investigated whether the release of FnIII‐1c due to mechanical forces and proteolysis can affect the differentiation of human MSCs via Wnt5a expression. We examined the involvement of Wnt5a in the differentiation induced by TLR4.

## 2 MATERIALS AND METHODS

### 2.1 Cell culture

hMSCs were acquired from the Stem Cell Bank of the Royan Institute in this study. The cells were cultured in α‐MEM (Gibco, Catalog No. 12571) supplemented with 10% FBS (Gibco, Catalog No. 10270106), and 1% penicillin/streptomycin (Sigma, Catalog No. 15‐070‐063). Cells in the 3rd passage were used for the experiments.

### 2.2 Multilineage differentiation potential of hMSCs

hMSCs were seeded at a density of 2 × 10^4^ cells/cm^2^ in a 6‐well plate with DMEM supplemented with 10% FBS, and 1% penicillin/streptomycin. After 24 hours, the basic medium was replaced with osteogenic and adipogenic differentiation medium with or without LPS 1 µg/mL (Invivogen, Catalog No. tlrl‐peklps) or FnIII‐1c 1 µg/mL (Sigma‐Aldrich, Catalog No. F3542). For chondrogenic differentiation, these ligands were added into pellets containing chondrogenic medium. Treatment was performed at the following time‐points: 7, 14 and 21 days.

For induction of osteogenic differentiation, DMEM supplemented with 10% FBS, 10 mM β‐glycerophosphate (Sigma‐Aldrich, Catalog No. 154804‐51‐0), 10^−8^ M dexamethasone (Sigma‐Aldrich, Catalog No. D4902) and 50 µg/mL ascorbic acid (Sigma‐Aldrich, Catalog No. A8960).

For induction of adipogenic differentiation, BM‐MSCs were cultured in adipogenic medium that contained DMEM supplemented with 10% FBS, 50 µg/mL indomethacin (Sigma‐Aldrich, Catalog No. 53‐86‐1), 50 µg/mL ascorbic acid and 1 × 10^−7^ M dexamethasone.

For chondrogenic differentiation, 15 mL polypropylene tube was used to seed 2 × 10^5^ cells and centrifuged at 250 *g* for 5 minutes at room temperature until pellets were formed. After centrifugation, the supernatant was gently removed and the pellets were cultured in chondrogenic medium, which contained DMEM as the basic medium supplemented with 10 ng/mL human transforming growth factor‐1 (Fitzgerald, Catalog No. 30R‐AT027), 10^−7^ M dexamethasone, 50 µg/mL ascorbic acid, 1% non‐essential amino acids (Gibco, Catalog No. 11140‐035) and 1% ITS (Gibco, REF. 41400‐045). The characterization of osteogenic, adipogenic and chondrogenic differentiation was performed by Alizarin red, Oil red O and toluidine blue/safranin‐O staining, respectively.

### 2.3 Alizarin red quantification

Alizarin red quantification was used for the evaluation of mineralization of the cells on exposure to FnIII‐1c at the following concentrations: 0.5, 1 and 1.5 µg/mL. First, the cells were fixed with 4% paraformaldehyde, and then Alizarin red was used for staining. Alizarin red quantification (Sigma‐Aldrich, Catalog No. ECM815) was performed according to the manufacturer's instructions by measuring absorbance at 405 nm in a microplate reader.

### 2.4 Alkaline phosphatase (ALP) activity

The effects of TLR4 ligands and TLR4 knockdown on the differentiation of BM‐MSCs into osteoblast cells were investigated as a function of ALP activity after 7, 14 and 21 days. An alkaline phosphatase assay kit (Colorimetric, Abcam, Catalog No. ab83369) was used to measure activity according to the manufacturer's protocol. The BCA protein (Novagen, Catalog No. 71285‐3) assay kit was used to determine the protein concentration. The specific activity was determined through enzyme activity normalized by total protein content.

### 2.5 RNA interference

The cells were seeded at a density of 2 × 10^5^ cells in 6‐well plates. Transfection of hMSCs was performed using psiRNA‐hTLR4 or psiRNA‐LucGL3 (control plasmid) (InvivoGen, Catalog No. ksirna42‐htlr4), and the lipofectamine 3000 (Invitrogen, Catalog No. L3000075) reagent according to the manufacturer's instructions. siRNA‐induced silencing of TLR4 gene was determined by Real‐time PCR and western blot techniques.

### 2.6 Real‐time PCR

To study the expression levels of genes involved in osteogenic, adipogenic and chondrogenic differentiation (For more details, see Table S1) as well as Wnt5a and TLR4, total RNA was extracted by TRI reagent (Sigma‐Aldrich, Catalog No. T9424) from cultured MSCs that were differentiated, and were untreated or treated with LPS/FnIII‐1c on days 0, 7, 14 and 21. To eliminate genomic DNA, RNAs were treated with DNase I (TAKARA, Catalog No. 2270A), according to the manufacturer's protocol. The PrimeScript™ RT reagent kit (TAKARA, Catalog No. RR037A) was used for cDNA synthesis from 1 μg total RNA. All real‐time polymerase chain reactions (PCR) were performed using SYBR Green Master Mix (Applied Biosystems Life Technologies, Inc, REF 4367659) and StepOnePlus Real‐time PCR System (Applied Biosystems Life Technologies, ABi) with the programme that was as follows: holding stage: 95°C (10 minutes), cycling stage: 95°C (15 seconds), 60°C (1 minutes) for 40 cycles. The melting curve stage was followed by a dissociation stage: 95°C (15 seconds), 60°C (1 minutes), and 95°C (15 seconds). β‐actin was used as an endogenous control to normalize all the target mRNAs. All biological groups were compared to the control group. The gene expression data analysis was carried out by 2^−ΔΔCT^ method. In Table S1, the sequence of the specific primers for each gene is listed.

### 2.7 Western blot analysis

The hMSCs transfected with psiTLR4 were washed twice with cold PBS and proteins were extracted using Mammalian Protein Prep Kit (QIAGEN, Catalog No. 37901) with protease and phosphatase inhibitors. The protein concentration was measured using the Bradford assay. Proteins were loaded on 10% SDS polyacrylamide gel and transferred onto PVDF membranes (Membrane Solutions). Non‐specific binding was blocked with 5% skim milk for 1 hr. The blocked membranes were incubated with anti‐TLR4 primary antibody (1:500, Abcam, Catalog No. ab22048) and β‐actin antibody (1:5000, Thermo Fisher Scientific, Catalog No. 60008‐1‐IG), then washed and incubated with horseradish peroxidase (HRP)‐conjugated secondary antibodies (1:30 000, Sigma‐Aldrich, Catalog No. 12‐349) for 1 hour at room temperature. Specific protein bands were identified by the ECL prime western blotting detection reagent (Amersham/GE Healthcare, Catalog No. RPN2232) and proteins were visualized by the chemiluminescence imaging system (UVITEC Cambridge).

### 2.8 Statistical analysis

Data were analysed using Prism statistical software (version 8). The statistical significance of the differences between groups was determined by one‐way analysis of variance (ANOVA) and followed by post hoc Tukey test. Also, the comparison between the two groups was done with *t*‐test. A *P*‐value of .05 was considered significant.

## 3 RESULTS

### 3.1 TLR4 expression increased during osteogenic, chondrogenic and adipogenic differentiation of hMSCs

**FIGURE 1:**
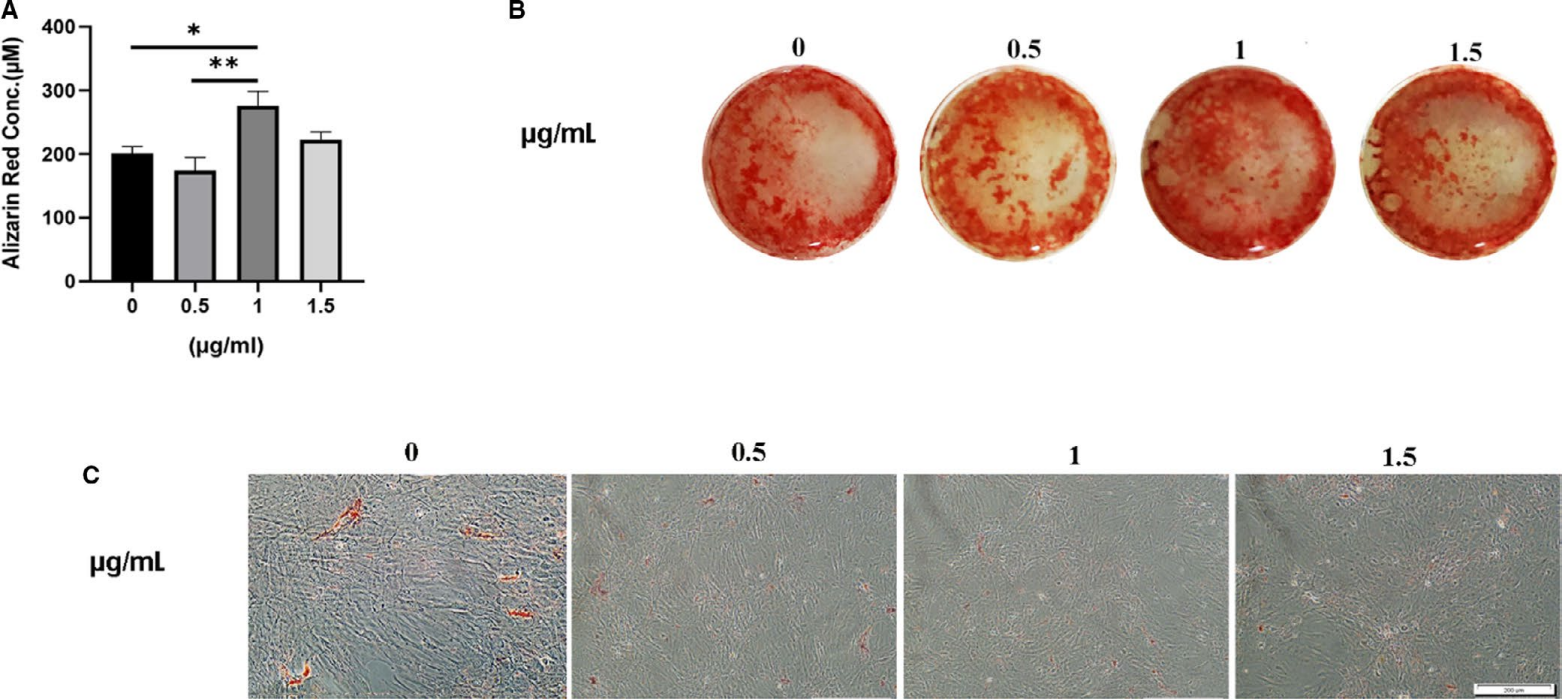
Effect of FnIII‐1c ligand on osteogenic and adipogenic differentiation of hMSCs. MSCs were cultured under different concentrations of FnIII‐1c in osteogenic and adipogenic media for 7 d, differentiation potential was evaluated through (scale bar = 200 mm) (A) Alizarin red quantification, (B) its staining and (C) Oil Red O staining in appropriate dose (0.5, 1 and 1.5 µg/mL)

**FIGURE 2:**
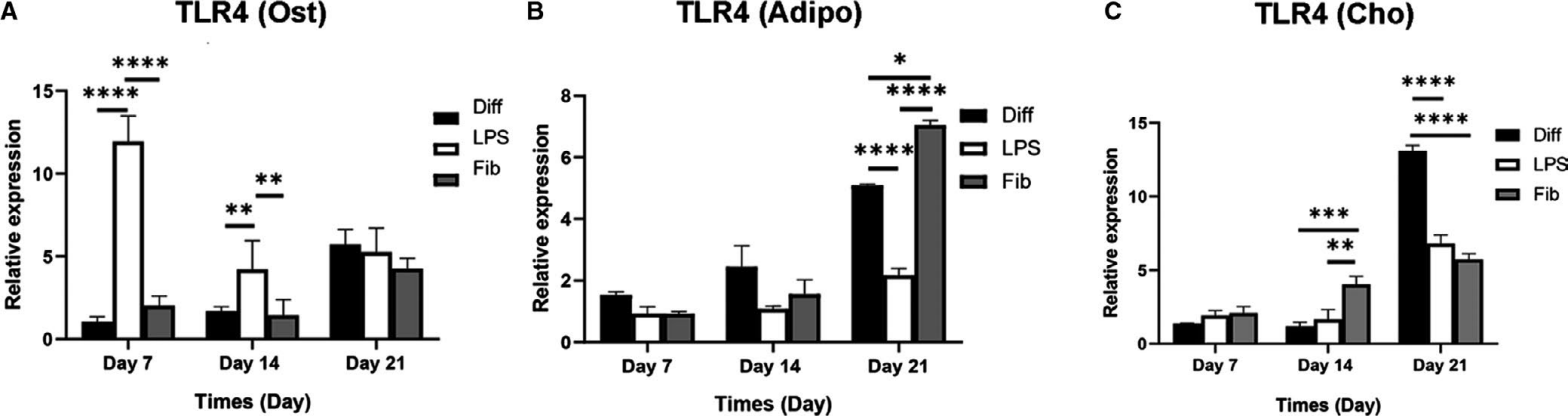
TLR4 expression during the differentiation of MSCs into the three respective lineages in the presence and absence of LPS or FnIII‐1c. MSCs were cultured for differentiation into three lineages then the cells were treated with and without 1 µg/mL LPS or FnIII‐1c for 7, 14 and 21 days. The mRNA levels of TLR4 were detected in cells cultured in (A) osteogenic, (B) adipogenic, and (C) chondrogenic media using real‐time PCR. Data are presented as mean ± SEM of ratios relative to β‐actin levels (n = 3). *P < .05, **P < .01, ***P < .001, ****P < .0001

To determine whether osteogenic, chondrogenic and adipogenic differentiation can affect TLR4 expression, human BM‐MSCs were cultured and differentiated into three different lineages using induction media for 7, 14 and 21 days. The cells also were treated with 1 µg/mL LPS, this concentration was selected based on a previous study.^23^, ^24^ To determine whether FnIII‐1c affects the osteogenic and adipogenic differentiation of MSCs, we added FnIII‐1c in an appropriate dose (0.5, 1 and 1.5 µg/mL) to the differentiation media of relevant at 7 days. The FnIII‐1c strongly stimulated the osteogenic differentiation of MSCs at a concentration of 1 µg/mL (based on alizarin red quantification and alizarin red staining), whereas it did not affect adipogenic differentiation (Oil red O staining). Therefore, a concentration of 1 µg/mL FnIII‐1c, was selected based on total results (Figure 1A‐C). TLR4 expression increased during osteogenesis, adipogenesis and chondrogenesis (Figure 2A‐C). LPS increased TLR4 expression on days 7 and 14 during osteogenesis significantly compared with the untreated differentiated group (Figure 2A). During adipogenesis, FnIII‐1c activation remarkably increased TLR4 expression on day 21 compared with the untreated group (Figure 2B). During chondrogenic differentiation, TLR4 expression on treatment with FnIII‐1c was significantly increased from day 14 (Figure 2C).

### 3.2 Both LPS and FnIII‐1c activation promoted osteogenic differentiation of hMSCs

**FIGURE 3:**
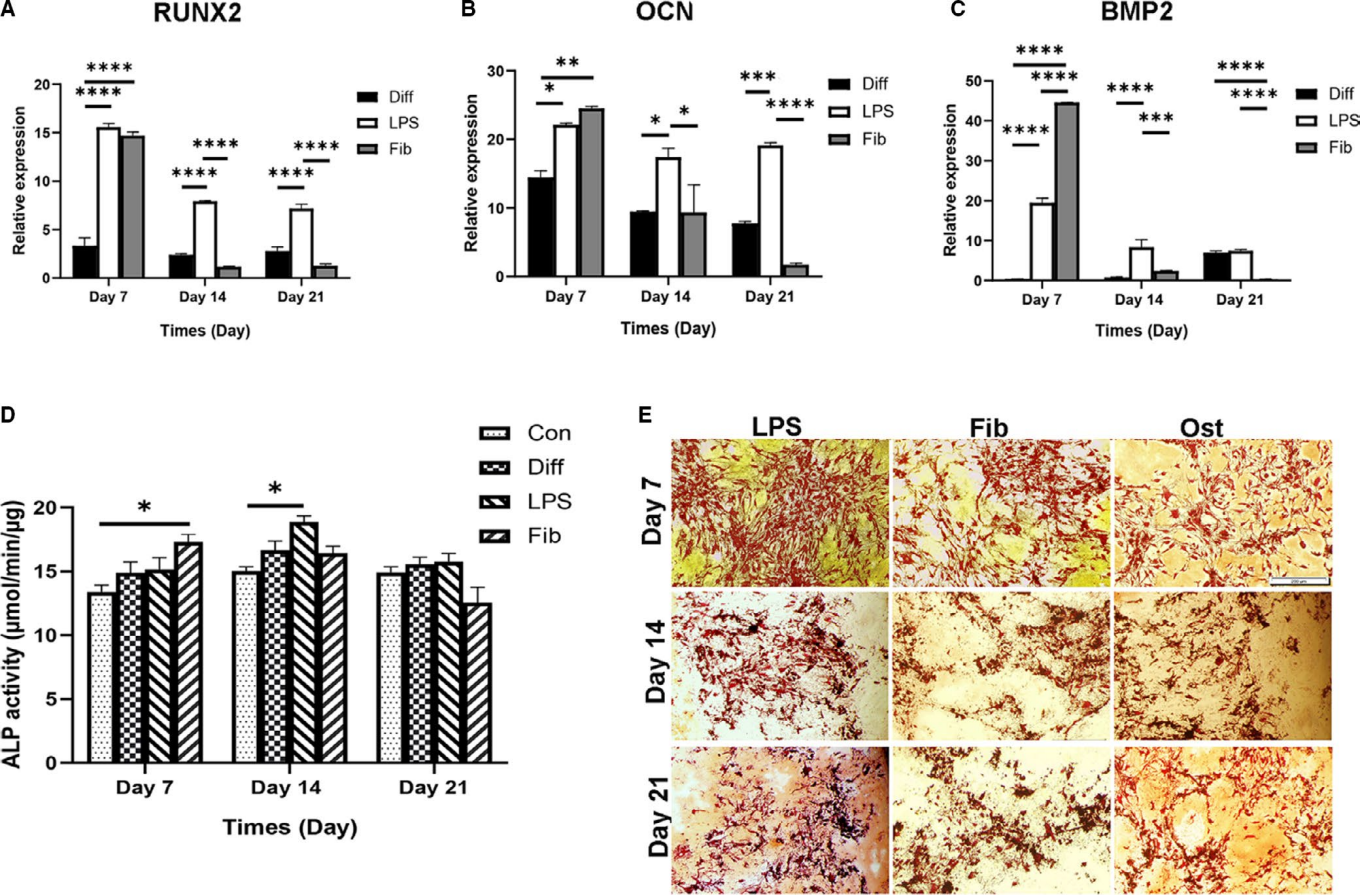
Effect of LPS and FnIII‐1c activation on the expression of genes involved in osteogenic differentiation of hMSCs. MSCs were cultured under osteogenic medium with and without 1 µg/mL LPS or FnIII‐1c for 7, 14 and 21 d for all the experiments mentioned below. The mRNA levels of (A) Runt‐related transcription factor 2, (B) osteocalcin, and (C), bone morphogenetic protein‐2 were evaluated using real‐time PCR. (D) ALP activity measurement and (E) characterization of hMSCs were conducted using Alizarin red staining (scale bar = 200 mm), MSCs were cultured in DMEM medium in the control group (Con). Data are presented as mean ± SEM of ratios relative to β‐actin levels (n = 3). *P < .05, **P < .01, ***P < .001, ****P < .0001

To detect whether TLR4 affects osteogenic differentiation differently upon exposure to its ligands, hMSCs were cultured in the absence or presence of 1 µg/mL LPS or FnIII‐1c in osteogenic media for TLR4 activation. ALP activity measurement and real‐time PCR were conducted for the assessment of osteogenesis in the MSCs at various time‐points. The levels of RUNX2, OCN and bone morphogenetic protein 2 (BMP2) on stimulation of FnIII‐1c on day 7 were higher than in the untreated group. RUNX2, OCN and BMP2 expression on days 7 and 14 on treatment with LPS compared with the untreated group were significantly enhanced (Figure 3A‐C). FnIII‐1c stimulation significantly decreased BMP2 expression in comparison with LPS and the untreated groups on day 21 (Figure 3C).

Assessment of osteogenesis by Alizarin red staining confirmed the formation of a mineralized matrix on days 7, 14 and 21 (Figure 3E). The results of ALP activity showed that FnIII‐1c affected the ALP activity remarkably on day 7 compared to the control group (Figure 3D) and ALP activity after LPS treatment was higher than in the control group on day 14.

### 3.3 TLR4 activation by LPS and FnIII‐1c decreased adipogenic differentiation of hMSCs

**FIGURE 4:**
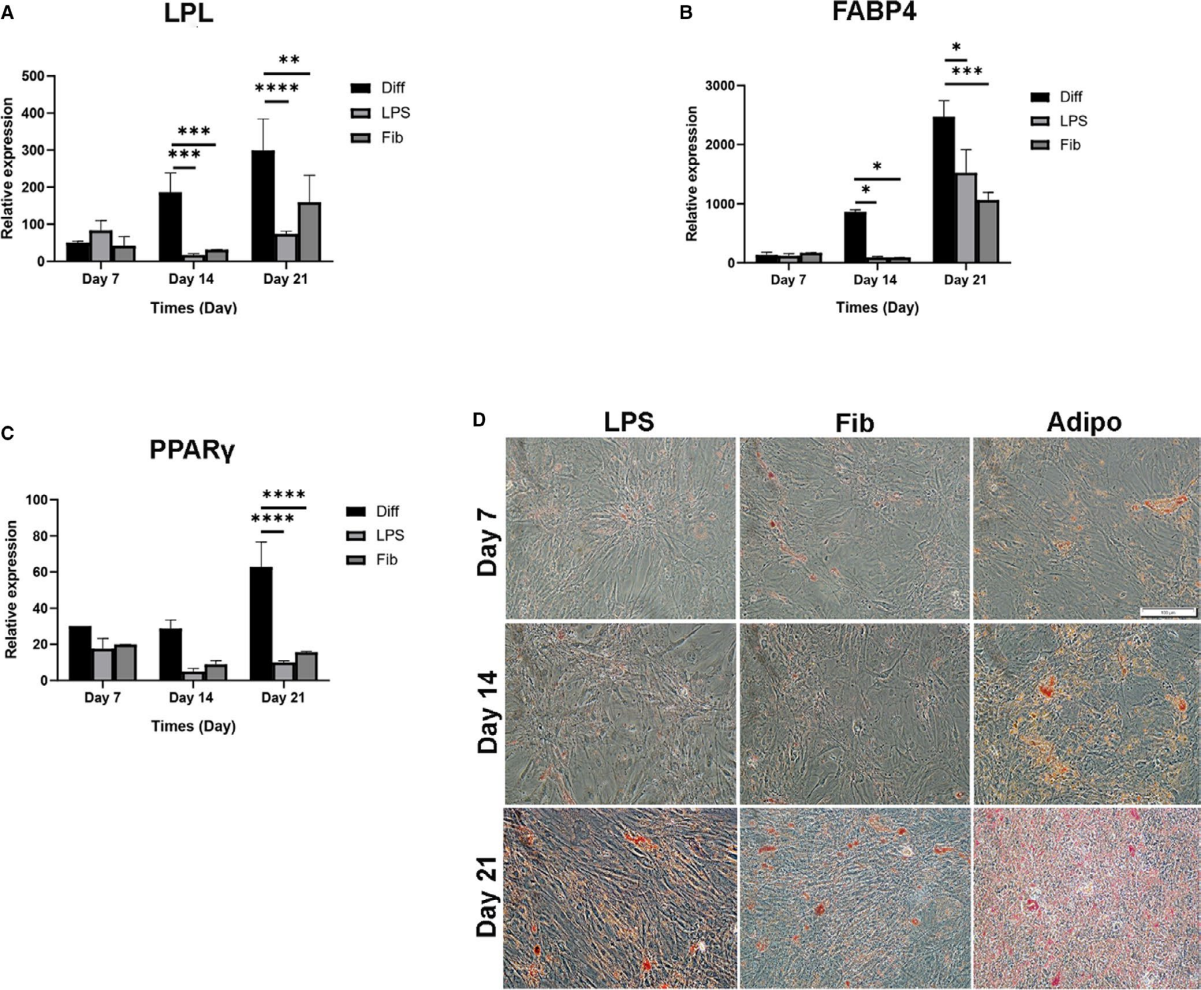
Effect of LPS and FnIII‐1c activation on the expression of genes involved in adipogenic differentiation of hMSCs. MSCs were cultured under adipogenic medium with and without 1 µg/mL LPS or FnIII‐1c for 7, 14 and 21 d for all the experiments mentioned below. The mRNA levels of (A) lipoprotein lipase, (B) Fatty acid‐binding protein 4 (FABP4), and (C) peroxisome proliferator‐activated receptor gamma (PPAR‐γ) were evaluated using real‐time PCR. (D) Characterization of hMSCs was conducted using Oil Red O staining (scale bar = 100 mm). Data are presented as mean ± SEM of ratios relative to β‐actin levels (n = 3). *P < .05, **P < .01, ***P < .001, ****P < .0001

We investigated whether the expression of relevant adipogenesis genes changed after adding endogenous and exogenous TLR4 ligands to MSCs cultured in adipogenic medium. The expression of FABP4, and LPL mRNA declined after LPS and FnIII‐1c treatment compared to the untreated differentiated group on days 14 and 21, and PPARγ mRNA expression significantly decreased only on day 21 (Figure 4A‐C). After 21 days, cells cultured in the presence of LPS and FnIII‐1c showed a staining density lower than that of the untreated group (Figure 4D).

### 3.4 TLR4 activation by LPS but not FnIII‐1c enhanced chondrogenic differentiation of hMSCs

**FIGURE 5:**
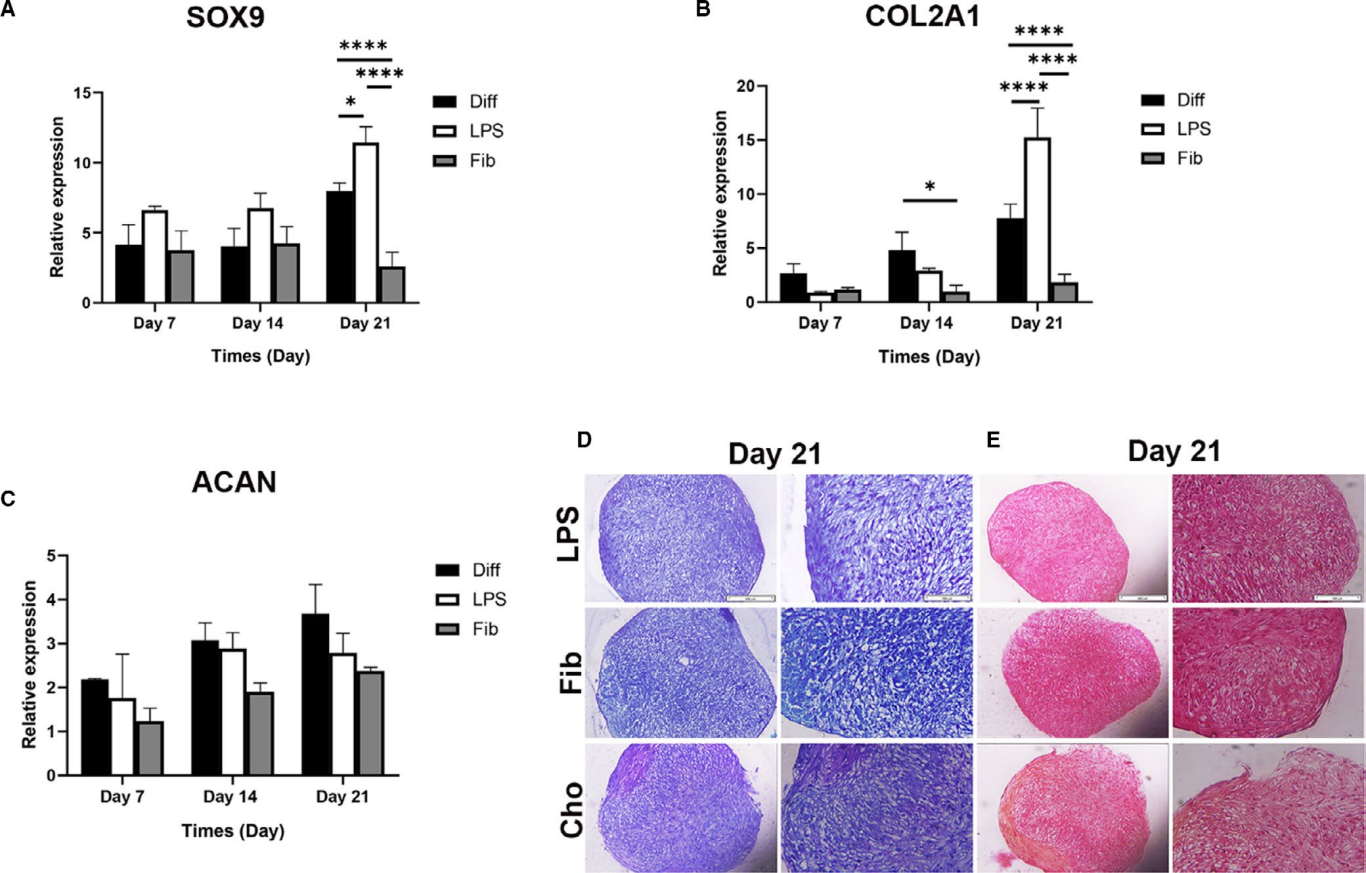
Effect of LPS and FnIII‐1c activation on the expression of genes involved in chondrogenic differentiation of hMSCs. MSCs were cultured in 15 mL polypropylene tube under chondrogenic medium with and without 1 µg/mL LPS or FnIII‐1c for 7, 14 and 21 d for all the experiments mentioned below. The mRNA levels of (A) SOX9, (B) type II collagen (COL2A1), and (C) Aggrecan (ACAN) was evaluated by real‐time PCR. Characterization of hMSCs was conducted using (D) toluidine blue, and (E) safranin‐O staining (scale bar = 100 and 200 mm respectively from the right in each column). Data are presented as mean ± SEM of ratios relative to β‐actin levels (n = 3). *P < .05, **P < .01, ***P < .001, ****P < .0001

To verify whether TLR4 endogenous and exogenous ligands affect the chondrogenic differentiation of hMSCs, LPS and FnIII‐1c were added to the chondrogenic medium. LPS stimulated the mRNA expression of SOX9 and COL2A1 compared to the untreated group on day 21 but FnIII‐1c activation strongly decreased expression of both SOX9 and COL2A1 compared to the untreated differentiated group (Figure 5A,B). After 21 days, the pellets that were cultured with and without LPS or FnIII‐1c were assessed for the chondrogenic phenotype using toluidine blue and Safranin‐O staining, the LPS and untreated groups had acquired a clear phenotype of differentiation in comparison with the FnIII‐1c group (Figure 5D,E).

### 3.5 Knockdown of TLR4 promotes adipogenesis, impairs osteogenesis and partially chondrogenesis of hMSCs

**FIGURE 6:**
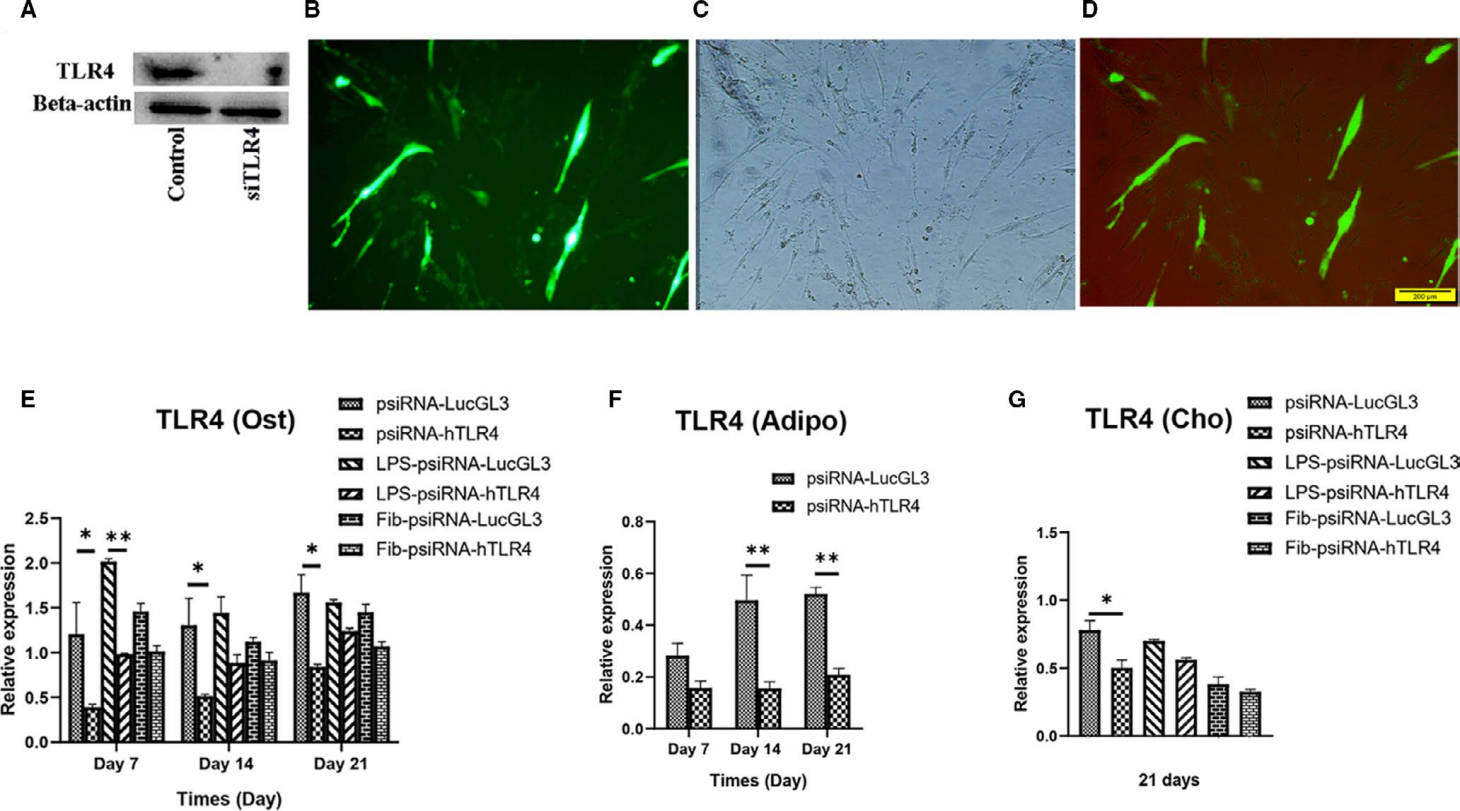
TLR4 silencing by psiTLR transfection. MSCs were transfected with psiTLR4 and psiRNA‐LucGL3 (control). (A) TLR4 levels in the TLR4 knockdown and control groups were detected through western blotting. MSCs were transfected with psiTLR4, transfection of hMSCs with psiTLR4 was confirmed using (B) fluorescence microscope, and (C) light microscope (Scale bar = 200 mm). (D) merged state is shown. TLR4 expression after knockdown of TLR4 in MSCs during (E) osteogenic, (F) adipogenic, and (G) chondrogenic differentiation was detected using real‐time PCR. Data are presented as mean ± SEM of ratios relative to β‐actin levels (n = 3). *P < .05, **P < .01, ***P < .001, ****P < .0001

**FIGURE 7:**
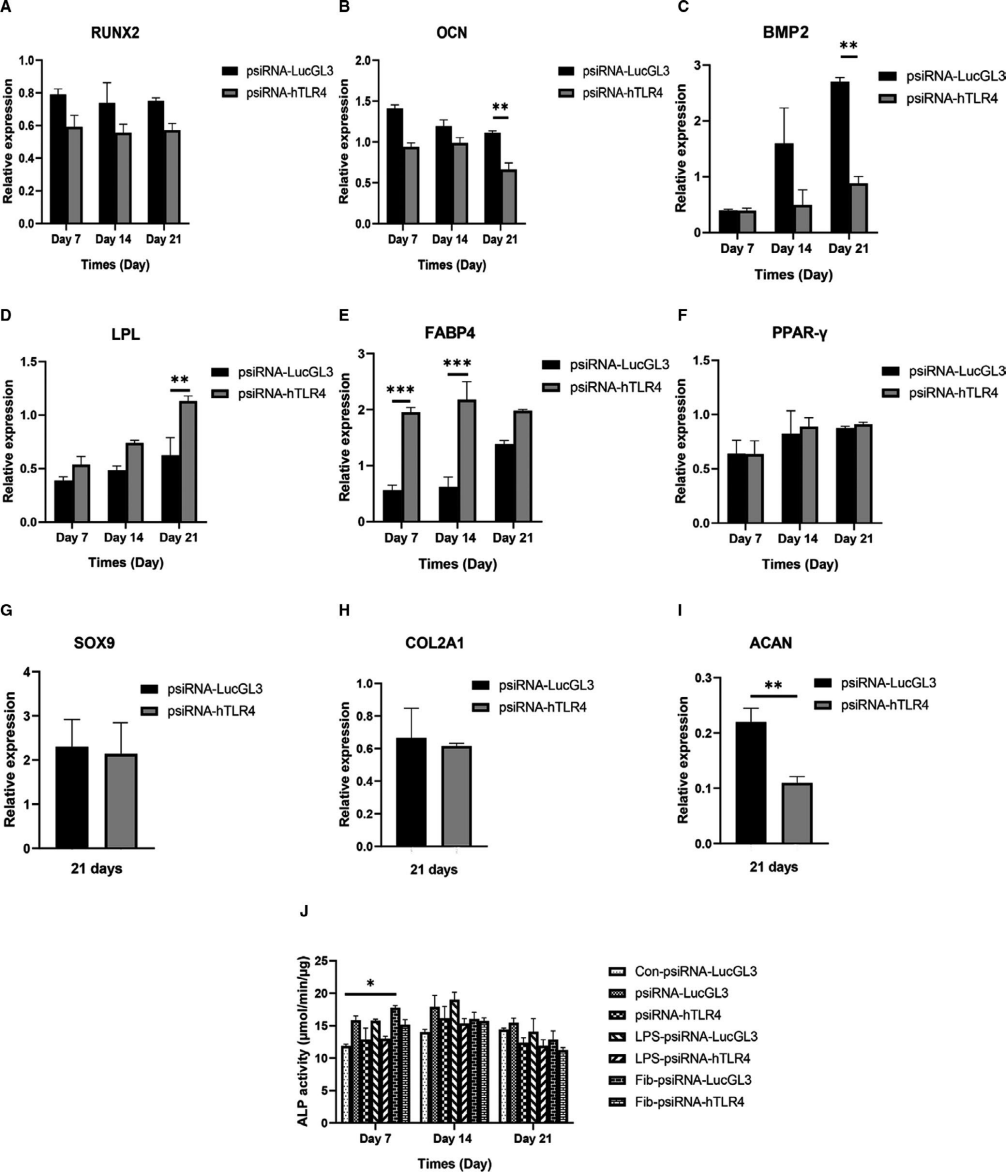
Effect of TLR4 knockdown on the expression of genes involved in osteogenic, adipogenic and chondrogenic differentiation. MSCs were transfected with psiRNA‐hTLR4 (SiTLR4 group) and psiRNA‐LucGL3 (control group). The mRNA levels of (A) Runt‐related transcription factor 2, (B) osteocalcin, (C) bone morphogenetic protein‐2, (D) lipoprotein lipase, (E) Fatty acid‐binding protein 4 (FABP4), (F) peroxisome proliferator‐activated receptor gamma (PPAR‐γ), (G) SOX9, (H) type II collagen (COL2A1) and (I) Aggrecan (ACAN) were evaluated using real‐time PCR for (A‐C) osteogenic (D‐F) adipogenic and (G‐I) chondrogenic differentiation, respectively. (J) ALP activity of treated groups undergoing osteogenic differentiation was evaluated, MSCs were cultured in DMEM medium in the control group (Con). Data are presented as mean ± SEM of ratios relative to β‐actin levels (n = 3). *P < .05, **P < .01, ***P < .001, ****P < .0001

psiRNA‐hTLR4 was used for knockdown of TLR4 in hMSCs. The knockdown of TLR4 was confirmed by mRNA expression and protein levels of TLR4 (Figure 6A‐G). Then, the effect of TLR4 knockdown on the differentiation potential of MSCs into osteogenic (Figure 7A‐C), adipogenic (Figure 7D‐F) and chondrogenic (Figure 7G‐I) lineages were evaluated by examining mRNA expression during differentiation. TLR4 expression was lower on LPS treatment of the TLR4 knockdown group compared to the LPS‐psiRNA‐LucGL3 group on day 7 (*P* < .01) (Figure 6E). TLR4 knockdown suppressed mRNA expression of OCN and BMP2 on day 21 during osteogenic differentiation (Figure 7B,C). In adipogenic differentiation, the knockdown of TLR4 increased FABP4 mRNA expression in differentiated hMSCs on days 7 and 14. Additionally, LPL expression in the TLR4 knockdown group was significantly higher than in the control group on day 21 (Figure 7D‐F). In chondrogenic differentiation, knockdown of TLR4 showed lower ACAN expression in comparison with the control group on day 21 (Figure 7I). ALP activity in the untreated group reached its maximum on day 14. The highest peak of ALP activity in the FnIII‐1c group was achieved on day 7. It was also observed that the TLR4 knockdown caused a decrease in ALP activity. Though ALP activity increased on treatment with LPS on day 7, the highest peak of activity was observed on day 14, and the TLR4 knockdown caused a decrease in activity here as well (Figure 7J).

### 3.6 LPS promoted Wnt5a expression during osteogenesis in a stage‐dependent manner and FnIII‐1c inhibited Wnt5a expression during adipogenesis

We analysed whether stress‐induced ligand (FnIII‐1c) can stimulate MSC differentiation into the three respective lineages in comparison with pathogen ligand (LPS). During osteogenic differentiation, Wnt5a expression increased in a time‐dependent manner, and LPS treatment significantly enhanced the Wnt5a level on day 7 (*P* < .0001) (Figure S1). Wnt5a expression upon FnIII‐1c activation significantly decreased during osteogenesis and adipogenesis on days 7, 14 and 21 in comparison with the untreated group (Figure S1). During chondrogenic differentiation, Wnt5a expression on treatment with and without LPS or FnIII‐1c reached a maximum on day 14 (Figure S1). During osteogenesis, Wnt5a expression was decreased in the TLR4 knockdown group in all three time‐points compared to in the control group, however, this difference was not significant (Figure S1). After knockdown of TLR4, there was no significant difference in Wnt5a levels between the control and TLR4 knockdown group upon adipogenesis induction (Figure S1). However, during chondrogenesis, upon TLR4 knockdown, Wnt5a expression was significantly higher than in the control group on day 21 (Due to differences between tested groups during chondrogenesis on day 21, the TLR4 knockdown effects were studied at this time‐point) (Figure S1).

## 4 DISCUSSION

The effect of TLR4 on the differentiation potential of MSCs is not clear. Our study for the first time showed that TLR4 expression increased under osteogenic, adipogenic and chondrogenic induction of MSCs. The stress‐induced ligand (FnIII‐1c) of TLR4 increased osteogenesis in the early stages of differentiation. TLR4 knockdown suppressed osteogenesis and partially chondrogenesis, however, it increased adipogenesis. Wnt5a expression was inversely related to the late stage of chondrogenesis in the process.

Our results show that the TLR4 expression was enhanced in hMSCs upon differentiation into the three lineages, hence osteoblasts, adipocytes and chondrocytes earned the capacity to enhance the expression of TLR4 during related differentiation. However, the baseline expression of TLR4 in MSCs helps to maintain self‐renewal properties. TLR4 expression upon LPS stimulation increased greatly in the early stages of MSCs differentiation into osteoblasts, then its expression gradually declined. Mo et al^23^ showed that long‐term exposure to LPS causes down‐regulation of TLR2 and TLR4 expression in osteoprogenitors. They observed that endotoxin negatively regulates TLR4 expression and this event is also observed in immune cells such as monocytes.^23^ This phenomenon was not observed in our study in either adipocytes or chondrocytes, it is likely that the event is cell‐type‐specific.

Transfer of LPS to the TLR4/MD2 complex is facilitated by LBP and CD14, but this transition of LPS to the molecular complex is a dynamic process required for the activation of the immune system.^25^ Therefore, this dynamic process may also affect the potential for the differentiation of the MSCs. In dermal fibroblasts, it has been shown that differences in the response of TLR4 to Tenascin C, FnIII‐1c or FnEDA may be due to differences in the co‐receptor that stimulate it. Therefore, the heterogeneity of the TLR4 receptor complex varies as a function of TLR4 activation.^17^

TLRs regulate many functions of MSCs upon stimulation by their ligands.^26^ LPS treatment did not affect the properties of immunosuppression and immunogenicity in human adipose‐derived stem cells (hASCs).^27^ Proliferation in mouse BM‐MSCs was enhanced following TLR4 stimulation.^28^ Recently, it has been shown that different responses occur on stimulation of TLR depending on the species (human or mouse) as well as the cell type of MSCs.^9^, ^29^, ^30^

Therefore, we evaluated whether a pattern of TLR4 expression is linked with the pattern of gene expression during the differentiation of human MSCs into the three respective lineages. In our study, both LPS and FnIII‐1c increased osteogenesis on day 7 sharply, however, ALP activity on treatment with FnIII‐1c reached its peak faster (on day 7) as compared to LPS activation (on day 14). Fibronectin probably released from injured sites can accelerate calcium deposition at the early stage of osteogenesis (on day 7) compared with LPS, which is an endotoxin (on day 14). This is important in the case of MSCs that transplanted for bone regeneration. Raicevic et al^31^ demonstrated that LPS (10 µg/mL) increased osteogenesis in ADSCs more than hMSCs, but it did not influence osteogenesis in Wharton's Jelly (WJ). Because of the constitutively low potential for osteogenesis in WJ, TLR4 activation did not affect it. Hwa Cho et al^32^ demonstrated that osteogenesis was enhanced after stimulation with osteogenic media treated with LPS in hADSCs. LPS (1 µg/mL) inhibited osteogenic and adipogenic differentiation compared to the control group in mouse MSCs.^33^ Recently, it has been shown that long‐term exposure to LPS (1 μg/mL and 10 μg/mL) can elevate calcium and ALP levels in MSCs on day 10. They suggested that the low expression of TLR4 is a mechanism of MSCs that protect from bacterial toxins and increases survival rates.^23^

Our results showed that chondrogenesis was increased by LPS in the late stage (on day 21) of differentiation whereas fibronectin inhibited it. This study for the first time showed that stimulation of the TLR4 by endogenous and exogenous ligands has different effects on the differentiation of MSCs into cartilage. Further research is required to focus on these findings.

Kim et al^34^ demonstrated that LPS increased the chondrogenic differentiation of UCMSCs for 3 weeks. One study revealed that TLR functionality is determined by a variety of pathways such as LBP and TGFβ1.^35^ Recently, a report showed that in addition to the role of TGFβ in the modulation of the immune system,^36^ it has a role in increasing collagen deposition in LPS‐primed MSCs while the deposition of fibronectin increased in poly(I: C)‐primed MSCs (a TLR3 ligand) which was associated with decreased levels of TGFβ.^37^ Therefore, the duration of exposure with a ligand, the type of ligand, the stage of differentiation, pathways of TLR stimulation (endogenous or exogenous), the concentration of ligand, the type and species of cells can affect the differentiation potential of MSC.

FnIII‐1c may affect different pathways and mechanisms involved in osteogenesis and chondrogenesis depending on the stage of differentiation and the gene network involved in each step. In a study, the probable cause of the limited effects of TLR4 stimulation by fibronectin, the effect of the endogenous ligand on several receptor classes in addition to the TLR4 in these cells were reported.^10^

In hMSCs, after stimulation by LPS, expression of IL‐1β, IL‐6 and tumour necrosis factor (TNF)‐a was enhanced but treatment with fibronectin led to an increase in the production of matrix metalloproteinase‐3 (MMP3).^10^ Production of IL‐6 was increased after a bone fracture in the inflammatory phases of fracture healing.^38^ In another study, LPS in addition to producing inflammatory cytokines, increased IP10 (CXCL10) expression that was activated via IFN1β, indicating that LPS has affected both MyD88‐dependent and MyD88‐independent pathways of hASCs.^39^ The MMPs play a role in ECM degradation, during chondrogenesis the levels of proteoglycans increased, which was associated with increased levels of genes related to ECM and decreased levels of MMP3.^40^ Overall it seems the production of cytokines upon TLR4 stimulation by LPS or fibronectin affects MSC fate into bone or cartilage.

To elucidate whether TLR4 is involved in MSC differentiation, we investigated if TLR4 knockdown affects the expression of genes involved in the differentiation into the three separate lineages of MSCs. Expression of osteogenesis‐related genes after TLR4 knockdown was decreased on day 21. LPS treatment during osteogenesis in the TLR4 knockdown group significantly decreased TLR4 expression in comparison to the LPS‐psiRNA‐LucGL3 group, to a lesser extent, in the fibronectin group.

TLR4 knockdown promoted adipogenesis in hMSCs. ACAN expression in the TLR4 knockdown group was significantly suppressed as compared to in the untreated group on day 21 indicating that chondrogenesis was partially suppressed. It also suggests the probability that pathways other than TLR4 are involved in chondrogenesis. In mice with MyD88‐deficient MSCs, upon stimulation of LPS induction of secretion of IL‐6 was not observed, osteogenesis and chondrogenesis were impaired, and they notably differentiate into adipocytes.^26^ Down‐regulation of MyD88 inhibited osteogenesis and adipogenesis in hASCs.^39^

The molecular mechanism of TLR4 in the differentiation of hMSCs into three lineages is still unknown. However, the MAPK pathway also plays a role in the osteogenesis of MSCs.^41^ Maximum ERK activation was observed during osteogenesis of hADSCs at day 7 which LPS stimulation enhanced.^32^ Stimulation of both LPS and fibronectin triggered the phosphoinositide 3‐kinase (PI3K) pathway, upstream of the MAPK and NF‐κB pathways in hMSCs.^10^ Recently, it has been shown that FnIII‐1c activates p38 MAP kinase and stable mRNA IL‐8.^17^ Therefore, it suggests that fibronectin may exert its effect on osteogenesis via MAPK.

Another pathway involved in the differentiation of MSCs is the Wnt5a pathway. TLRs and NF‐kB pathways play a role in Wnt5a expression.^42^ In human dental pulp stem cells, Wnt5a expression increased due to LPS via the pathways of TLR4/MyD88, NF‐kB or PI3K/AKT.^43^ It has been shown that TLRs alone are not enough for the regulation of the expression of Wnt5a but it was suggested that particular requirements for cell type and condition of differentiation could also affect.^42^

These results raise the possibility that TLR4 may play a diverse role in regulating Wnt signalling in different tissues. Overexpression of Wnt5a via the promoter of osteopontin drives osteogenesis in MSCs and suppressing Wnt5a promoted differentiation of MSCs into pre‐adipocytes.^44^ In our study, up‐regulation of Wnt5a expression was observed during osteogenesis and adipogenesis and it seems that Wnt5a stimulation by LPS increases osteogenesis on day 7. Fibronectin is released in unfavourable conditions under mechanical stimulation in damaged tissues, through up‐regulated Wnt5a which could not increase osteogenesis. There was a difference in Wnt5a expression between the TLR4 knockdown (lower) and the control group, however, it was not significant in osteogenesis. Wnt5a expression in the tested groups increased until day 14 during chondrogenesis and then decreased on day 21. A study demonstrated that Wnt5a expression was promoted in the early stages of chondrogenesis via SOX9 by the induction of calmodulin kinase or the nuclear factor of activated T cells (NFAT).^45^ In the stages of early chondrogenesis, Wnt5a suppresses the canonical pathway for differentiation into cartilage, however, it induces the non‐canonical pathway which contributes to the destruction of cartilage.^46^ Therefore, it seems that Wnt5a plays a role in the onset of chondrogenic differentiation. To determine definitively the effect of Wnt5a on the differentiation of mesenchymal stem cells, the effect of Wnt5a siRNA on the differentiation of hMSCs in the absence and presence of TLR4 agonists should be determined.

## 5 CONCLUSION

We observed that TLR4 expression increased during the differentiation of MSCs into all three lineages. However, the basic expression of TLR4 is the mechanism that preserves the self‐renewal capacity of MSCs. We also revealed that TLR4 knockdown suppresses osteogenesis while promoting adipogenesis in human MSCs. Our data clarify that the FnIII‐1c promotes osteogenesis in the early stages of differentiation but it decreased chondrogenesis in the late stage of the process.

Up‐regulation of TLR4 has a direct link with enhanced osteogenesis and chondrogenesis depending on the type of ligand and the stage of differentiation but its expression was inversely related to adipogenesis. This study elucidates how the presence of stress or pathogens alters TLR4 expression and if the differentiation potential of MSCs is affected, which is important to comprehend from a clinical or treatment perspective.

## CONFLICT OF INTEREST

The authors confirm that there are no conflicts of interest.

## AUTHOR CONTRIBUTION

**Fatemeh Khodabandehloo:** Investigation (lead); Writing‐original draft (lead). **Reza Aflatoonian:** Investigation (supporting); Visualization (lead). **Zahra Zandieh:** Investigation (supporting); Methodology (supporting). **Farzad Rajaei:** Investigation (supporting). **Forugh‐Azam Sayahpour:** Investigation (supporting). **Marjan Nassiri‐Asl:** Funding acquisition (equal); Supervision (equal); Writing‐review & editing (lead). **Mohamadreza Baghaban Eslaminejad:** Funding acquisition (equal); Supervision (equal); Writing‐review & editing (equal).

## Supporting information

Supplementary Material

## Data Availability

The data are available from the corresponding author.
