# Supplementary material for: Functional differences of Toll‐like receptor 4 in osteogenesis, adipogenesis and chondrogenesis in human bone marrow‐derived mesenchymal stem cells
Source: J Cell Mol Med. 2021 May 3;25(11):5138–49. doi: 10.1111/jcmm.16506 (PMC8178267; doi:10.1111/jcmm.16506)
Supplement: Supplementary file 1 — Supplementary Material [file JCMM-25-5138-s001.docx]

**Supplementary information**

**Supplementary Table S1.** primer sequences used for real‐time PCR analysis.

| **Target Gene** | **Primer Sequence** | **Accession number** | **Product Size** |
| --- | --- | --- | --- |
| TLR4 | TGATGTCTGCCTCGCGCCTG  AACCACCTCCACGCAGGGCT | NM_138554.5 | 98bp |
| Beta-actin | CAAGATCATTGCTCCTCCTG  ATCCACATCTGCTGGAAGG | NM_001101.5 | 90bp |
| Wnt5a | TCCTTCGCCCAGGTTGTAATTG  AGTGGCACAGTTTCTTCTGTCC | NM_001256105.1 | 151bp |
| Osteocalcin(OCN) | GTGCAGAGTCCAGCAAAGGT  TCAGCCAACTCGTCACAGTC | [NM_199173.6](https://www.ncbi.nlm.nih.gov/entrez/viewer.fcgi?db=nucleotide&id=1519313061) | 175bp |
| RUNX2 | ATGACACTGCCACCTCTGA  ATGAAATGCTTGGGAACTGC | NM_001024630.3 | 117bp |
| BMP2 | GGAGGCAAAGAAAAGGAACGGA  GAAGCAGCAACGCTAGAAGACA | NM_001200.4 | 175bp |
| COL2A1 | ACTCAAGTCCCTCAACAACC  ATCCAGTAGTCTCCACTCTTCC | NM_033150.3 | 126bp |
| SOX9 | CCCTTCAACCTCCCACACTAC  GCTGTGTGTAGACGGGTTGTT | NM_000346.3 | 253bp |
| Aggrecan (ACAN) | CTGGACAAGTGCTATGCCG  GAAGGAACCGCTGAAATGC | NM_001135.3 | 191bp |
| LPL | TCAACTGGATGGAGGAGGAG  GGGGCTTCTGCATACTCAAA | NM_000237.3 | 169bp |
| PPARγ | TCTCCAGCATTTCTACTCCACA  GATGCAGGCTCCACTTTGAT | NM_138712.3 | 127bp |
| FABP4 | CAGTGTGAATGGGGATGTGA  GGACACCCCCATCTAAGGTT | NM_001442.3 | 146bp |

**Supplementary Figure S1**


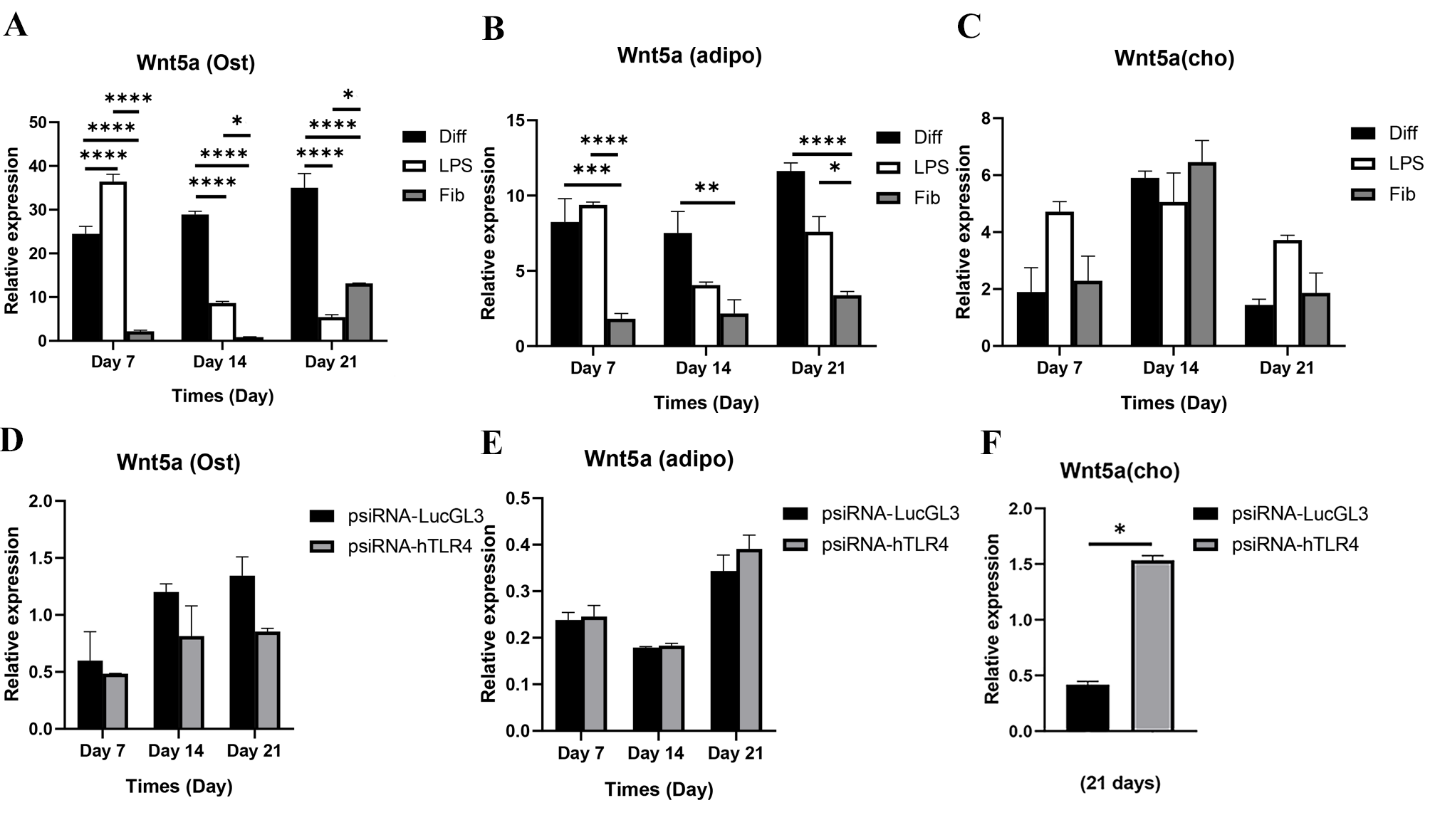


**Figure S1** Wnt5a expression after TLR4 knockdown and during the differentiation of MSCs into osteogenic, chondrogenic, and adipogenic lineages. Wnt5a expression was evaluated in treated MSCs with and without LPS or FnIII-1c during (A) osteogenic, (B) adipogenic, and (C) chondrogenic differentiation. MSCs were transfected with psiRNA-hTLR4 (SiTLR4 group) and psiRNA-LucGL3 (control group), then the mRNA levels of Wnt5a during (D) osteogenic, (E) adipogenic, and (F) chondrogenic differentiation days using real-time PCR were evaluated. Data are presented as mean ± SEM of ratios relative to β-actin levels (n = 3). *P < 0.05**P < 0.01***P < 0.001****P < 0.0001.
